# ﻿A new species of *Lophostreptus* Cook, 1895 discovered among syntypes of *L.regularis* Attems, 1909 (Diplopoda, Spirostreptida, Spirostreptidae)

**DOI:** 10.3897/zookeys.1188.115802

**Published:** 2024-01-10

**Authors:** Henrik Enghoff, Nesrine Akkari

**Affiliations:** 1 Natural History Museum of Denmark, University of Copenhagen, Universitetsparken 15, DK-2100 Copenhagen OE, Denmark University of Copenhagen Copenhagen Denmark; 2 Naturhistorisches Museum Wien, Burgring 7, 1010 Wien, Austria Naturhistorisches Museum Wien Wien Austria

**Keywords:** Kilimanjaro, millipedes, natural history museums, shelf-life, Tanzania, types

## Abstract

A new species of the genus *Lophostreptus* Cook, 1895 is described, based on specimens hidden for over a century among the syntypes of its congener *Lophostreptusregularis* Attems, 1909 housed in the Naturhistoriska Riksmuseet Stockholm (NRMS) and the Naturhistorisches Museum Wien (NHMW). A lectotype is designated for *Lophostreptusregularis* Attems, 1909 in order to stabilize its taxonomy. Updates to the millipede fauna of Mt. Kilimanjaro, Tanzania are provided.

## ﻿Introduction

It quite often happens that re-examination of type material of species described by previous authors reveals that the type series includes several species. A striking myriapod example is the centipede *Lithobiuslapidicola* Meinert, 1872, the type material of which turned out to include no less than nine species (Eason 1974). We are not aware of similarly impressive millipede cases, but there are several examples of millipede type series which have subsequently been shown to include more than one species. In some of these cases the type series of a species includes an additional undescribed species. Thus, Demange (1981) described *Carlogonusverhoeffi* Demange, 1981 (Harpagophoridae) from a specimen found in the presumed type series of *Indiothaumajonesi* Verhoeff, 1938, and Golovatch et al. (2017a) described *Annaminaattemsi* Golovatch, Geoffroy & Akkari, 2017 (Paradoxosomatidae) from a specimen found in the type series of *A.xanthoptera* Attems, 1937.

We here put on record one more such example and describe a new species found among syntypes of *Lophostreptusregularis* Attems, 1909. This nominal species, described from Mt. Kilimanjaro in Tanzania, has long been regarded a junior subjective synonym of *L.ptilostreptoides* Carl, 1909, also from Tanzania. Recently, Enghoff et al. (in press) confirmed the suspicion of Carl (1909), viz., that his aptly named *L.ptilostreptoides* is the same as *Ptilostreptustersus* Cook, 1896, now valid as *Lophostreptustersus* (Cook, 1896). Enghoff et al. (in press) examined syntypes of *L.regularis* belonging to Naturhistorisches Museum Wien (NHMW) and Naturhistoriska Riksmuseet Stockholm (NRMS) and found that one alcohol-preserved male from NRMS, as well as a set of gonopods on a slide from NHMW, belonged to an undescribed species.

In this paper we describe this new species, provide information on type material of *L.regularis*, select a lectotype of *L.regularis* in order to avoid future confusion, and give updates to the list of millipedes from Mt. Kilimanjaro by Enghoff and Frederiksen (2018).

## ﻿Material and methods

The type material was obtained from Naturhistoriska Riksmuseet Stockholm (NRMS) and Naturhistoriches Museum Wien (NHMW), studied and photographed in NHMW using a Nikon DS-Ri2 camera mounted on a Nikon SMZ25 stereomicroscope or Nikon Eclipse, using NIS-Elements Microscope Imaging Software with an Extended Depth of Focus (EDF). Obtained images were edited in Adobe Photoshop 2024 and assembled in Adobe InDesign 2024. Symbols used in the description are explained in the text and in the figure legends.

## ﻿Results

### ﻿Taxonomy

**Class Diplopoda de Blainville in Gervais, 1844**

**Order Spirostreptida Brandt, 1833**

**Family Spirostreptidae Brandt, 1833**

#### ﻿Genus Lophostreptus

Taxon classificationAnimaliaSpirostreptidaSpirostreptidae

Cook, 1895

AA7E91A8-EDE4-5325-AF19-6CED5F937EF3

##### Type species.

*Glyphijulusmagnus* Karsch, 1881, by original designation. Male not known.

##### Diagnosis.

A trachystreptoform (sensu Enghoff et al. in press) genus with the anterior margin of collum unmodified, the lateroapical metaplical process (*lap*) of the gonopod coxa inclined or abruptly bent laterad, and the generally slender gonopod telopodite carrying a platelike post-torsal extension (*sf*) or at least with a marked ‘knee’ (*kn*) at the same place (from Enghoff et al. in press).

##### Other included species.

*Lophostreptusarmatus* Pocock, 1896.

*Lophostreptusbicolor* Carl, 1909.

*Lophostreptuscameranii* Silvestri, 1896. Male not known.

*Lophostreptusluridus* Attems, 1934. Male not known.

*Lophostreptusmagombera*Enghoff et al. (in press).

*Lophostreptusminimus* Mwabvu & VandenSpiegel, 2009.

*Lophostreptusporiger* Verhoeff, 1941. Male not known.

*Lophostreptussimilis* Attems, 1914.

*Lophostreptustersus* (Cook, 1896).

*Lophostreptusulopygus* Attems, 1928. Male not known.

#### ﻿ Lophostreptus tersus

Taxon classificationAnimaliaSpirostreptidaSpirostreptidae

(Cook, 1896)

B795E2CF-469B-52B0-9DA1-A4E28665891F

Ptilostreptus tersus Cook, 1896: 57.
Lophostreptus ptilostreptoides Carl, 1909: 321, synonymized by Enghoff et al. (in press) [synonymy tentatively suggested by Carl (1909: 317)].
Lophostreptus regularis Attems, 1909: 31, synonymized with L.ptilostreptoides by Krabbe (1982: 258).
Lophostreptus tersus : Attems (1914: 143).
Lophostreptus malleolus Kraus, 1958: 12, synonymized with L.ptilostreptoides by Demange and Mauriès (1975: 79).
Lophogonus ptilostreptoides : Demange and Mauriès (1975: 78).

##### Material examined.

***Lectotype* of *L.regularis*** (NHMW MY8871) 1 slide with a pair of gonopods, one telopodite broken off. “1. 2. Bp cf” “Lophostreptus/ regularis/ Kibonoto” [leg. Sjöstedt Y., 1905–1906, & don. Sjöstedt/ Attems]”. Lectotype here designated. ***Paralectotypes* of *L.regularis* NHRS**:1 ♂, 6 ♀♀; Kilimandjaro, Kibonoto, Stepp-Kulturzon; 1000–1900 m a.s.l.; Oct. 1905; Y. Sjöstedt leg.; also a second ♂ of a different species, see below (NHRS-TOBI 000005480); 4 ♀♀; Kilimandjaro, Kibonoto, Massaistäppen; 1000 m a.s.l.; 23 Aug. 19905; Y. Sjöstedt leg. (NHRS-TOBI 000005478); 1 ♀; Usambara, Tanga, Jun. 1905; Y. Sjöstedt leg. (NHRS-TOBI-000005482); 4♀♀; Kilimandjaro, Kibonoto; Nov. 1905; Y. Sjöstedt leg.; under multnande blad I bananfarmerkulturzon (under decaying leaves in banana farm cultural zone) (NHRS-TOBI-000005476); 4♀♀; Kilimandjaro, Kibonoto; 1300 m a.s.l.; 1905; Y. Sjöstedt leg. I förnan under nedfallna plantanblad (in förna [plant litter] under fallen plantain leaves) (NHRS-TOBI-000005479);1 ♀; Kilimandjaro, Kibonoto; 1905; Y. Sjöstedt leg.; Mischwald – Kulturzone (NHRS-TOBI-000005481); 4♀♀, 4 anamorphic juv.; Kilimandjaro, Kibonoto; Nov. 1905; Y. Sjöstedt leg.; Kulturzon (NHRS-TOBI-0000077). **NHMW**: 1 ♀ anterior body section, 1 ♀, 4 ♀♀ anterior body sections, 5 posterior body sections, 5 middle parts, 3 vials: head, collum & body segments; head & body segments (labelled “♂”); head, collum & body sections (labelled “♀”); 1 slide: three pairs of legs, antenna, gnathochilarium. Tanzania, Kilimanjaro region, Hai district, Steppe, cultivated area, mixed forest 1000–1900 m “1) Lophostreptusregularis Att/ Kilimandjaro, Kibonoto/ Steppe – Kulturzone 1000–1900 m/ Sjöstedt” (NHMW MY2466); 2 ♀♀, 2 ♀♀ anterior body sections, 3 juveniles, 1 juvenile anterior body section, three middle parts, three posterior body sections. Tanzania, Kilimanjaro region, Hai district, “Kibonoto” under rotten leaves, banana plantations “2) Lophostreptusregularis/ Kilimandjaro. Kibonoto/ In Farmen unter ver-/ faulten blättern/ Sjöstedt” (NHMW MY2467); slides “Lophostreptus/ regularis/ Kibonoto”, “Sjöstedt”: 1) “♂ [note in shorthand]” two pairs of legs and three single legs, 2) gonopods, one is dissected, 3) “♂ 3. 4. Sg” two segments, 4) “♀ 2. 1. 3. Bp” gnathochilarium and three pairs of legs, 5) “♀♀(KOH)” parts of segments (NHMW MY 4076). **MfN**: 10 specimens in several fragments. Although the type material is supposed to include 2 males, 5 females and 3 juveniles, we were not able to find any male among the specimens.

##### Remarks.

This species was discussed at length by Enghoff et al. (in press). Because the type material of *Lophostreptusregularis* contains two species, we here designate a lectotype of this nominal species although it is regarded as a junior synonym of *L.tersus*. We also provide details of the type material of *L.regularis* kept in NHMW. Further paralectotypes are housed in Naturhistoriska Riksmuseet Stockholm (NRMS) and Museum für Naturkunde Berlin (MfN) (see list of material) – to our knowledge, there are no syntypes of this species in any other collection.

#### ﻿ Lophostreptus neglectus sp. nov.

Taxon classificationAnimaliaSpirostreptidaSpirostreptidae

926B7C3F-50F8-5636-BE29-CDF831C6C6B9

https://zoobank.org/2BF66A14-5F02-4BEB-AE55-72AF29565ABF

Figs 1 , 2 , 3

##### Diagnosis.

Differing from all other species of *Lophostreptus* of which the male characters are known (see Remarks) by the shape of the distal part of the gonopod coxa.

##### Etymology.

Named ‘neglectus’ (adjective) because this species remained neglected despite a slide containing its gonopods in Attems’ type material (Fig. 3C).

##### Material examined.

***Holotype*.** ♂, Tanzania, Kilimandjaro, Kibonoto, Stepp-Kulturzon; 1000–1900 m a.s.l.; Oct. 1905; Y. Sjöstedt leg., NHRS-TOBI 000005630; separated from sample of 2 ♂♂, 6 ♀♀; syntypes (now paralectotypes) of *Lophostreptusregularis* (NHRS-TOBI 000005480). ***Paratype*.** Tanzania, Kilimanjaro region, Hai district, “Kibonoto”, syntype of *Lophostreptusregularis*NHMW MY10381, slide “6” (ex NHMW MY 4076). “1. 2. Bp cf Kilimandjaro” gonopods and two pairs of legs. “Kibonoto” [leg. Sjöstedt Y., 1905–1906, & don. Sjöstedt/ Attems]”.

##### Description

**(holotype male). *Size*.** Length 33 mm; midbody vertical diameter 2.4 mm; 46 podous rings, no apodous rings in front of telson.

**Figure 1.**
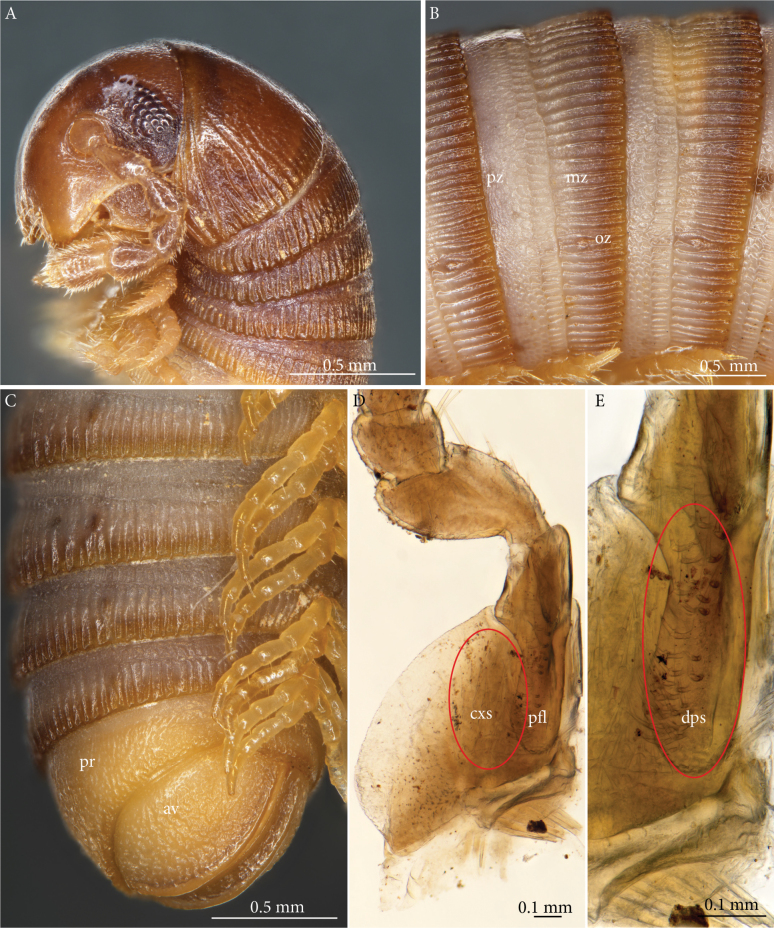
*Lophostreptusneglectus* sp. nov., holotype (NHRS-TOBI 000005630) **A** head, collum and rings 2–4, lateral view **B** midbody rings, lateral view **C** posterior rings with legs, and telson, (ventro-)lateral view **D** left leg of 1^st^ pair, anterior view **E** the same, close-up of prefemoral lobe. For **A–C**, the specimen was superficially dried and returned to alcohol after photography. Abbreviations: *av* = anal valve, *cxs* = coxosternal setae, *dps* = setae of prefemoral lobe, *mz* = metazona, *oz* = ozopore, *pfl* = prefemoral lobe, *pr* = preanal ring, *pz* = prozona.

***Colour*** (Fig. 1A–C). After more than 100 years in alcohol overall light brown, with a darker hue along metazonital keels, posterior part of metazonites amber. Telson and legs yellowish.

***Head*** (Fig. 1A). Vertex densely punctuate, with a clearly demarcated parietal furrow. Eyes not reaching mesal of antennal socket, c. 22 ommatidia in 5–6 horizontal and c. 8 vertical rows. Antennae reaching 3^rd^ body ring. Antenno­meres 3–5 strongly narrowed at base.

***Collum*** (Fig. 1A). Not modified for accommodation of antennae, coarsely punctate; along the posterior margin a row of quite short, weak furrows and carinae which towards the sides gradually reach further forwards. Lateral lobes much narrower than the dorsal part, not expanded, traversed by 3 anteriorly strongly ascending carinae/furrows of which the uppermost is the strongest and almost straight, reaching anterior margin above eye level, anterior corner rectangular, posterior corner more rounded, margins straight.

***Body rings*** (Fig. 1B). Prozonites (*pz*) in anterior part (c. half) with very fine ring furrows which further back give place to a cell structure; posterior part (c. 20%) delimited by clear line, with a regular pattern of larger, rectangular cells. The cuticular scutes (‘cytoscutes’) of the anterior part of the prozonite are remarkable by being rounded rather than polygonal and by being arranged in an imbricate pattern, as described for several other trachystreptoform species by Enghoff et al. (in press, e.g., fig. 12G, left inset). Suture between pro- and metazonites straight, simple. Metazonites (*mz*) with faint constriction a little behind suture, with numerous simple keels which reach from suture, across constriction and until posterior ring margin; c. 20 keels between dorsal midline and ozopore. Ozopores (*oz*) small, a little in front of middle of metazonite. Sigilla not seen.

***Telson*** (Fig. 1C). Preanal ring (*pr*) regularly and densely grainy-rugose. Anal valves (*av*) overall with same sculpture, strongly vaulted, their mesal margins slightly raised as low rims, smooth, meeting in midline, paralleled more laterally by much higher lips with smooth edge; lips higher than distance between lips and mesal margin; area between mesal margin and lip with weaker sculpture than main part of valve.

***Legs*.** Short, length c. 0.6× body diameter. No ventral pads. First pair (Fig. 1D, E): coxosternum with a few lateral setae (not evident in Fig. 1D), mesally with large groups of numerous long setae (*cxs*) next to prefemoral lobes. Prefemoral lobes (*pfl*) parallel-sided, c. twice as long as broad, apically broadly rounded, with a field of long setae (*dps*) extending from tip of process almost to its base.

**Figure 2.**
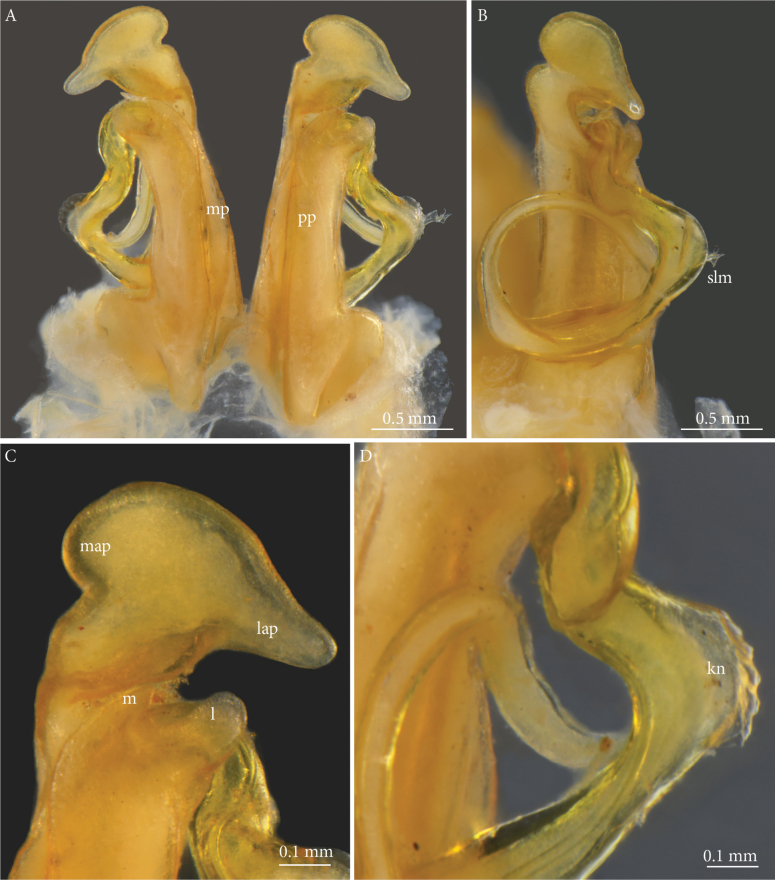
*Lophostreptusneglectus* sp. nov., holotype (NHRS-TOBI 000005630), gonopods **A** anterior view **B** left gonopod, posterior-lateral view **C** right gonopod, apical part, anterior view **D** telopodite. Abbreviations: *kn* = ‘knee’, *l* = lateral proplical process, *lap* = lateroapical process, *m* = mesal proplical process, *map* = mesapical process, *mp* = metaplica, *pp* = proplica, *slm* = solenomere.

***Gonopod coxa*** (Fig. 2). Proplica (*pp*) parallel-sided, apically curving slightly laterad and with a subsemicircular incision separating a broadly rounded lateral process (*l*) from a triangular mesal one (*m*). Metaplica (*mp*) with straight, only very slightly converging margins, hence almost same width throughout, subapically with mesal incision delimiting a smoothly rounded apical part with a semicircular mesapical process (*map*) and a relatively short, straight, tongue-shaped lateroapical process (*lap*).

**Figure 3.**
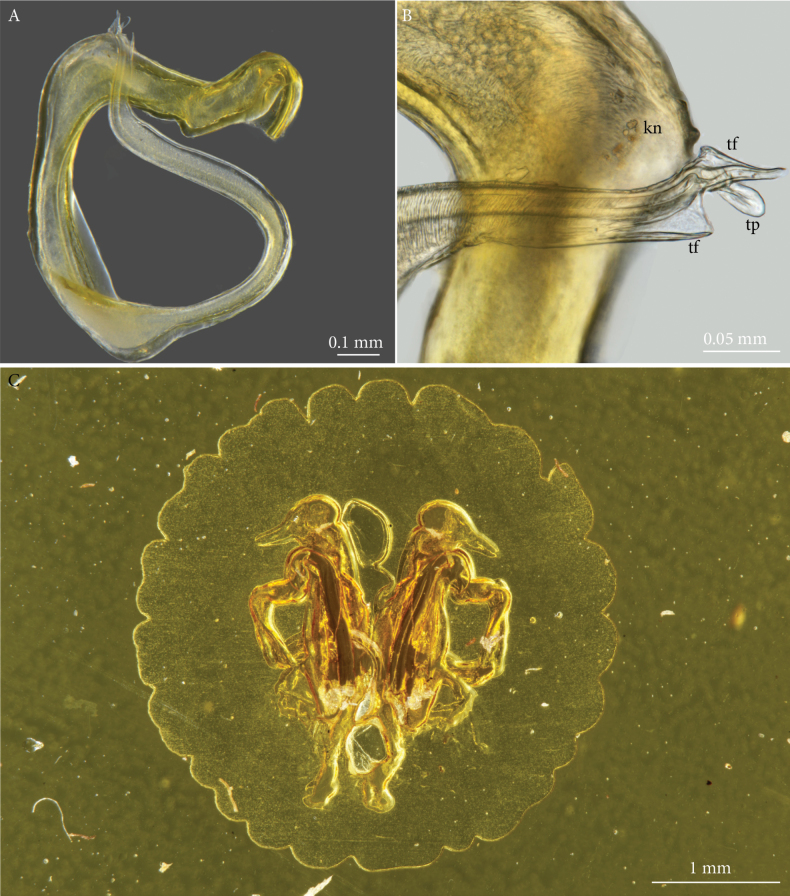
*Lophostreptusneglectus* sp. nov. **A**, **B** holotype (NHRS-TOBI 000005630) **A** left gonopod telopodite, except basal part **B** distal part of telopodite, showing details of solenomere **C** paratype (NHMW MY10381). Abbreviations: *kn* = ‘knee’, *tf* = triangular flange, *tp* = tongue-shaped process.

***Gonopod telopodite*** (Figs 2A, B, D, 3A, B). Slender, simple, with a knobby lateral ‘knee’ (*kn*) shortly after the emergence of the telopodite from the gonocoel, thereafter forming a full circle. Solenomere (*slm*) flanked by two triangular flanges (*tf*) and a tongue-shaped process (*tp*).

##### Descriptive notes on paratype.

The paratype consists of a complete set of gonopods. Despite the overall poor condition of the slide, these gonopods are obviously identical to those of the holotype.

##### Remarks.

The gonopods of *L.neglectus* sp. nov. are clearly different from those of the other *Lophostreptus* species of which the male is known. The remaining species which are currently assigned to *Lophostreptus*, but which – due to the lack of gonopod information – may just as well belong to one or more other ‘trachystreptoform’ genera, all seem to be bigger and/or derive from localities far away from Mt. Kilimanjaro.

## ﻿Discussion

*Lophostreptusneglectus* sp. nov. clearly belongs to the genus *Lophostreptus* as currently (Enghoff et al. in press) understood, but future much-needed phylogenetic analyses of Spirostreptidae may well change our conception of this genus. For now, *Lophostreptusneglectus* sp. nov. should be regarded as an endemic for Mt. Kilimanjaro, and with the addition of this species, the list of millipedes known from Mt. Kilimanjaro includes 38 species. Since the review by Enghoff and Frederiksen (2018), there have been a few other changes to the list: ‘Prionopetalum n. sp. cf. aculeatum Attems, 1914’ has been described as *Prionopetalumnessiae* Rosenmejer & Enghoff, 2021 (Rosenmejer and Enghoff 2021), ‘*Lophostreptusptilostreptoides* Carl, 1909’ is now *Lophostreptustersus* (Cook, 1896) (Enghoff et al. in press), ‘*Proxenodesmus* n. sp.’ has been re-identified as *Rhododesmusmastophorus* (Gerstäcker, 1873) (HE, unpublished), and ‘*Procoptodesmusdiffusus* Brolemann, 1920’ has been transferred to the genus *Cryptocorypha* Attems, 1907 (Golovatch et al. 2017b).

## Supplementary Material

XML Treatment for Lophostreptus

XML Treatment for Lophostreptus tersus

XML Treatment for Lophostreptus neglectus

## ﻿Additional information

### Author ORCIDs

Henrik Enghoff https://orcid.org/0000-0002-2764-8750

Nesrine Akkari https://orcid.org/0000-0001-5019-4833

### Data availability

All of the data that support the findings of this study are available in the main text
